# Brazilian family with hyperferritinemia-cataract syndrome: case report

**DOI:** 10.31744/einstein_journal/2022RC0076

**Published:** 2022-10-13

**Authors:** Aline Morgan Alvarenga, Nathália Kozikas da Silva, Rodolfo Delfini Cançado, Luís Eduardo Morato Rebouças de Carvalho, Paulo Caleb Junior Lima Santos

**Affiliations:** 1 Escola Paulista de Medicina Universidade Federal de São Paulo São Paulo SP Brazil Escola Paulista de Medicina, Universidade Federal de São Paulo, São Paulo, SP, Brazil.; 2 Faculdade de Ciências Médicas Santa Casa de São Paulo São Paulo SP Brazil Faculdade de Ciências Médicas da Santa Casa de São Paulo, São Paulo, SP, Brazil.

**Keywords:** Ferritins, Cataract, Hyperferritinemia, Mutation, Iron overload, Iron metabolism disorders

## Abstract

Hereditary hyperferritinemia-cataract syndrome is a rare autosomal dominant disease caused by a genetic mutation in the iron responsive element in the 5’ untranslated region of the ferritin light chain gene. Hereditary hyperferritinemia-cataract syndrome is characterized by elevated serum ferritin levels and bilateral cataract development early in life and may be misdiagnosed as hemochromatosis. This case report describes a Brazilian family with a clinical diagnosis of hereditary hyperferritinemia-cataract syndrome, which was submitted to ferritin light chain gene sequencing. The genetic mutation c.-164C>G was identified in the 5’ untranslated region. In conclusion, genetic testing can be used for accurate diagnosis of hereditary hyperferritinemia-cataract syndrome to avoid misdiagnosis of hemochromatosis, other diseases associated with iron overload or ophthalmic diseases.

## INTRODUCTION

Hereditary hyperferritinemia-cataract syndrome (HHCS) is a rare autosomal dominant disease^(1)^ characterized by elevated serum ferritin levels and bilateral cataract development early in life.^(2,3)^ Hereditary hyperferritinemia-cataract syndrome is caused by a genetic alteration in the iron-responsive element in the 5’ untranslated region (UTR) of the *ferritin light chain* (*FTL*) gene.^(4)^ The *FTL* gene is located in chromosome 19q13, contains four exons and encodes the light subunit of the ferritin protein. The 5’UTR region of the *FTL* and iron-regulating proteins are involved in the regulation of intracellular iron concentration.^(5-10)^ Rather than a single mutational origin, several mutations of the *FTL* gene can trigger HHCS.

At least 25 different genetic alterations, including the whole iron responsive element structure, have been reported in families with HHCS. Sporadic cases due to *de novo* mutations have also been described.^(11,12)^ Some of these genetic alterations interfere with the loop that interacts directly with iron regulatory proteins, while others affect the stems or the bulge of the iron-responsive element structure and modify its conformation.^(13,14)^

Specific genetic mutations in the iron-responsive element prevent it from binding to iron regulatory proteins, leading to continuous *FTL* gene synthesis and elevation of ferritin levels, with no impact on transferrin saturation (TS). This case report describes a Brazilian family with a clinical diagnosis of HHCS. Exon sequencing of the *FTL* gene was performed and revealed the presence of the genetic mutation c.-164C>G in the 5’UTR region.^(14)^

### Study protocol

Four primer pairs were designed to explore the 4 exons (including an important 5’UTR region) of the *FTL* gene, extending to 20bps of intronic regions. Polymerase chain reaction (PCR) was carried out and the product purified with Exo SAP-IT (GE Healthcare, NJ, USA). Next, a reaction was prepared for Sanger sequencing using Applied Biosystems 3130/3130xl Genetic Analyzers. After sequencing, the electropherogram was analyzed using the Geneious software (Biomatters).^(15)^

**Figure 1:**
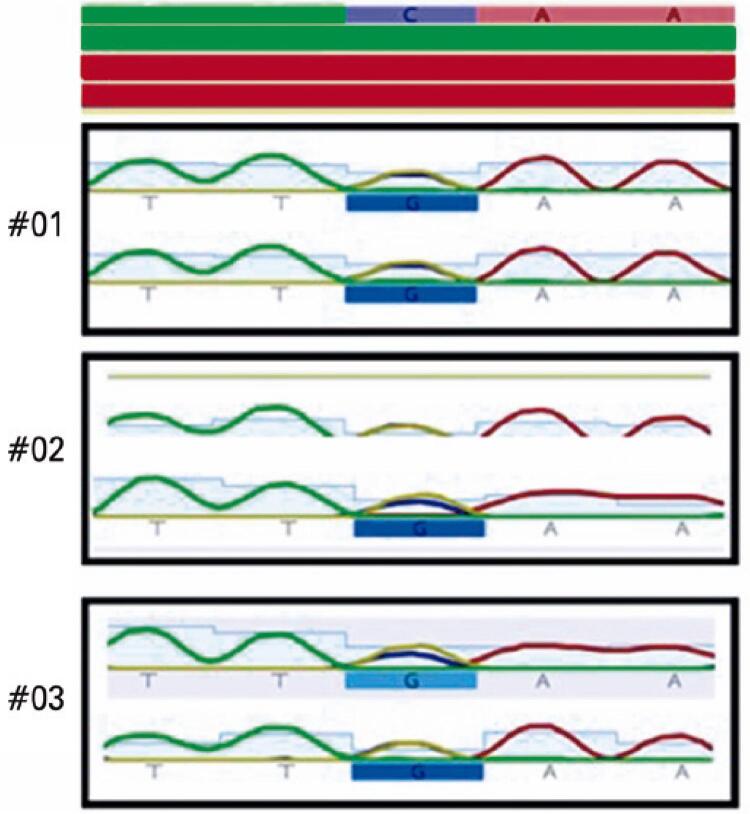
Ferritin light chain gene sequencing results. Patients #01, #02, #03: heterozygous genotype for the c.-164C>G pathogenic mutation

Sequencing of the 5’UTR region of the *FTL* gene revealed the pathogenic c.-164C>G mutation (Figure 1), first described in 2006, in all three family members.^(16)^

The Ethics Committee of *Escola Paulista de Medicina* (EPM)*, Universidade Federal de São Paulo* approved the study protocol (CAAE: 01507518.5.0000.5505; # 3.439.573). The participants signed a consent form prior to entering the study.

## CASE REPORT

A 45-year-old Brazilian female patient living in São Paulo, Brazil, was referred to our service in 2016. Her first laboratory tests revealed iron levels of 100mcg/dL and serum ferritin (SF) levels of 1,487ng/mL and plasma TS of 28%. No signs of hepatic iron overload were seen on magnetic resonance imaging and her liver iron concentration was 1.1mg/g. Echocardiogram and abdominal ultrasound findings were unremarkable. She was HBsAg- and anti-HCV-negative. Genetic analysis failed to detect the pathogenic mutations p.Cys282Tyr and p.His63Asp in the *HFE*. Tests were repeated in 2020, with the following results: iron level of 80mcg/dL, SF level of 1,434ng/mL and TS of 33%. The second magnetic resonance imaging report described absence of hepatic iron overload, liver iron concentration of 1.0mg/g and mild hepatic steatosis. The reason she had sought treatment was a personal and family history of decreased visual acuity and glare. She had been diagnosed with bilateral cataract at the age of 25 years. Progressive glare prompted bilateral cataract surgery when she was in her fifties. In patients with HHCS, cataracts may develop early in infancy^(17)^ and have been described as several dust-like lens opacities.^(18)^

The mother of the index case is 72 years old. In 2016, she had the following laboratory test results: SF of 1,703ng/mL and TS of 46%. She was also HBsAg- and anti-HCV-negative. Tests were repeated in 2020 and revealed iron level of 105μg/dL, SF level of 2,145ng/mL and TS of 34%. There was no evidence of iron overload on magnetic resonance imaging and her hepatic iron concentration was 1.3mg/g. No pathogenic mutations (p.Cys282Tyr and p.His63Asp) were found in the *HFE* gene.

In 2016, the son of the index case (an 11-year-old boy) had the following laboratory test results: iron level of 105μg/dL, SF level of 1,260ng/mL and TS of 28%. In 2020, his tests revealed iron level of 176μg/dL, SF level of 1,308ng/mL and TS of 46%. Magnetic resonance imaging showed no evidence of iron overload, and his hepatic iron concentration was 0.8mg/g. No pathogenic mutations (p.Cys282Tyr and p.His63Asp) were found in the *HFE* gene.

The proband and both family members were submitted to ophthalmic examination. Specific clinical and ophthalmological examinations revealed they all had cataracts.

## DISCUSSION

The prevalence of HHCS in Brazil is unknown. However, in Australia the estimated prevalence is around 1/200,000. The clinical manifestations of HHCS are congenital cataract and persistent hyperferritinemia unrelated to iron overload. Congenital cataract is associated with several diseases. Hence, investigation of *FTL* gene mutations is seldom included in the diagnostic workup. Most patients affected by this syndrome are evaluated due to hyperferritinemia. *HFE* gene mutations associated with hemochromatosis are common in the Brazilian population,^(19)^ and are the primary diagnostic hypothesis in these patients, leading to unnecessary diagnostic procedures and therapeutic phlebotomies.^(20)^

The frequency and *in silico* prediction of the pathogenic mutation in the population and the classification according to American College of Medical Genetics and Genomics (ACMG) criteria are shown in table 1. The mutation detected was assigned the “disease causing” status according to mutation Taster and was classified as “pathogenic” in ClinVar. As to others *in silico* predictors, no results were found. The mutation was also classified as “pathogenic” according to the ACMG.

**Table 1:** General patient information, clinical data, and classification of alterations according to American College of Medical Genetics and Genomics criteria

ID	Sex/Age	TS (%) / and SF (μg/L) 2016	TS (%) / and SF (μg/L) 2020	Liver iron concentration (mg/g) 2020	*FTL* variants	ACMG criteria	ACMG
#01	Female/41	28/1,487	33/1,434	1.0	c-164 C>A	PS, PM, PP	Pathogenic
#02	Female/67	46/1,703	34/2,145	1.3	c-164 C>A	PS, PM, PP	Pathogenic
#03	Male/11	28/1,260	46/1,308	0.8	c-164 C>A	PS, PM, PP	Pathogenic

ID: identification; TS: transferrin saturation; SF: serum ferritin; PS: pathogenic strong; PM: pathogenicity moderate; PP: pathogenicity supplementary; *FTL*: ferritin light chain; ACMG: American College of Medical Genetics and Genomics.

The genetic mutation c.-164C>G detected in the family described had been previously reported by another Brazilian group. That group described a case of a male Caucasian patient aged 7 years presenting with elevated serum ferritin and hemoglobin levels. The patient was tested for the pathogenic hemochromatosis mutation and for hepatitis, with negative results. The pathogenic mutation was also found in his mother.^(20)^

After analysis of the index case, the remaining cases (both index case relatives) could be screened. Laboratory tests revealed increased ferritin synthesis regardless of iron levels. The diagnosis was based on findings of clinical and specific examinations for cataract detection. The *FTL* gene was also sequenced and revealed the presence of the pathogenic genetic mutation in all three patients. This family is being followed by a medical team and is frequently examined, apart from having received medical guidance.

Patients with HHCS may be misdiagnosed with hemochromatosis, liver dysfunction or inflammation. Some patients may be submitted to invasive and unnecessary diagnostic procedures, such as liver biopsy, and may be inappropriately treated with phlebotomies, which may cause severe iron deficiency anemia. Hence the importance of accurate clinical and genetic diagnosis. Accurate diagnosis also contributes to earlier prescription of appropriate treatment.^(21)^

## CONCLUSION

Genetic testing can be used for accurate diagnosis of hereditary hyperferritinemia-cataract syndrome to prevent misdiagnosis of hemochromatosis, other diseases associated with iron overload or ophthalmic diseases.
